# Intrusion-Aware Alert Validation Algorithm for Cooperative Distributed Intrusion Detection Schemes of Wireless Sensor Networks

**DOI:** 10.3390/s90805989

**Published:** 2009-07-28

**Authors:** Riaz Ahmed Shaikh, Hassan Jameel, Brian J. d’Auriol, Heejo Lee, Sungyoung Lee, Young-Jae Song

**Affiliations:** 1 Department of Computer Engineering, Kyung Hee University, Suwon, Korea; E-Mails: riaz@oslab.khu.ac.kr (R.A.S.); daurial@oslab.khu.ac.kr (B.J.D.); yjsong@khu.ac.kr (Y.J.S.); 2 Computing Department, Macquarie University, NSW, Australia; E-Mail: hasghar@science.mq.edu.au; 3 Department of Computer Science & Engineering, Korea University, Seoul, Korea; E-Mail: heejo@korea.ac.kr

**Keywords:** alerts, anomalies, intrusions, trust management, wireless sensor networks

## Abstract

Existing anomaly and intrusion detection schemes of wireless sensor networks have mainly focused on the detection of intrusions. Once the intrusion is detected, an alerts or claims will be generated. However, any *unidentified* malicious nodes in the network could send faulty anomaly and intrusion claims about the legitimate nodes to the other nodes. Verifying the validity of such claims is a critical and challenging issue that is not considered in the existing cooperative-based distributed anomaly and intrusion detection schemes of wireless sensor networks. In this paper, we propose a validation algorithm that addresses this problem. This algorithm utilizes the concept of intrusion-aware reliability that helps to provide adequate reliability at a modest communication cost. In this paper, we also provide a security resiliency analysis of the proposed intrusion-aware alert validation algorithm.

## 1. Introduction

Many anomaly and intrusion detection schemes (IDS) have been proposed for wireless sensor networks (WSNs) [1–6], but those schemes mainly focus on the detection of malicious or faulty nodes. All those anomaly and intrusion detection schemes (IDS) that are cooperative in nature [1, 2, 4] need to share anomalies or intrusion claims with the other node(s). However, those schemes are unable to ascertain that the alert or claim received by the other node(s) is in fact sent by the trusted node(s). As a result, any *unidentified* malicious node(s) in the network could send faulty anomaly and intrusion claims about the legitimate node(s) to the other node(s). Verifying the validity of such claims is a critical issue that is not considered in existing cooperative-based distributed anomaly and IDS schemes of WSNs [7]. Recently, some intrusion prevention schemes that are based on alerts have been proposed in the literature [8, 9]. However, these schemes are based on the assumption that the monitoring nodes are trusted or the claim will be trusted if the monitoring node passed simple authentication and integrity test based on shared pair-wise key.

In this paper, we propose a new intrusion-aware alert validation algorithm that provides a mechanism for verifying anomaly and intrusion claims sent by any unidentified malicious node(s). This algorithm is simple and easy to implement. Our proposed algorithm execute on alert sender monitoring nodes and alert receiver monitoring nodes. Sender monitoring nodes are mainly responsible for the detection of malicious nodes, assignment of threat level, and generation of alert messages, whereas receiver monitoring nodes are mainly responsible for the validation of alert messages. Validation mechanism consists of two phases: consensus phase and decision phase. Although the consensus approach is widely used in distributed computing domain to solve many problems like fault-tolerance [10], here we used this approach with variation to solve problem of trusting anomaly and intrusion claims. In consensus phase, we uniquely introduce an intrusion-aware reliability concept that helps to provide an adequate reliability at a modest communication cost. In the decision phase, a node will make the decision regarding validation and invalidation of a claim based on the result of consensus phase.

The rest of the paper is organized as follows: Section 2 contains description on taxonomy of IDS. Section 3 describes related work. Section 4 discusses the network model, assumptions and definitions. Section 5 describes the proposed validation algorithm. Section 6 provides the analysis and evaluation of proposed algorithm in terms of communication overhead, reliability and security. Finally, Section 7 concludes the paper and highlights some future work.

## 2. Taxonomy of IDS

**Figure 1.**
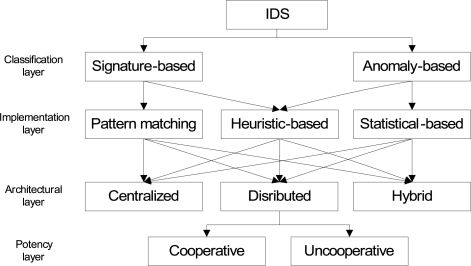
Taxonomy of intrusion detection schemes.

From the classification point of view, IDS have often been categorized into two types: signature-based IDS and anomaly-based IDS as shown in Figure 1. The signature-based IDS schemes (mostly implemented via pattern matching approach) detect intrusions based on the attack’s signature, such as, specific byte sequence in the payload or specific information in the header fields like sender address, last hop address, etc. On the other hand, the anomaly-based IDS (mostly implemented via statistical approach), first determines the normal network activity and then checks all traffic that deviates from the normal and marks it as anomalous.

In order to strengthen the signature-based and anomaly-based IDS schemes, some researchers applied heuristic algorithms. Heuristic approaches are generally used in AI. Instead of looking for exact pattern matches or simple thresholds, heuristic-based IDS “looks for behavior that is out of ordinary” [11] during specific time interval. In simple words, it “uses an algorithm to determine whether an alarm should be fired” [12]. For example, if a threshold number of unique ports are scanned on a particular host or a specific attack pattern signature is detected, then alarm will be fired [12].

From an architectural point of view, IDS schemes are further categorized into three categories: centralized, distributed and hybrid. In the centralized approach, a single designated node monitors the whole network. In the distributed approach, every node or a group of nodes monitor the network. In the hybrid approach, every group has one selected primary node responsible for monitoring and detecting anomalies and intrusions. Once the information is gathered, it is forwarded to the central base station which calculates the impact of those anomalies and intrusions on the whole network.

From the potency point of view, distributed approach is further classified into cooperative and unco-operative distributed approaches. In the cooperative distributed approach, every node or a group of nodes exchanges information about the anomalies and intrusions in order to detect collaborative intrusion attacks. On the contrary, in the uncooperative distributed approach, nodes do not share information about anomalies and intrusion with each others.

## 3. Related Work

### 3.1. Intrusion Detection Schemes

Intrusion detection schemes are not in itself the main focus of this paper. However, in order to give a brief overview of those, we have summarized the existing proposed anomalies and IDS schemes of WSNs in Table 1, in which [1, 2, 4, 6] are distributed and cooperative in nature. Brief descriptions of some of the proposed schemes are given below.

Bhuse *et al.* [1] have proposed different lightweight techniques for detecting anomalies for various layers, such as application, network, MAC and physical. The main advantage of the proposed techniques is the low overhead that makes them energy efficient. This is due to the fact that they reuse the already available system information (e.g., RSSI values, round trip time, etc.) which are brought forth at various layers of network stack.

Chatzigiannakis *et al.* [4] have proposed an application level anomaly detection approach that fuses data (comprised of multiple metrics) gathered from different sensor nodes. In the proposed scheme, the authors have applied Principal Component Analysis (PCA) to reduce the dimensionality of a data set. So this approach will help to detect correlated anomalies/attacks that involve multiple groups of sensors.

Du *et al.* [2] have proposed a localization anomalies detection (LAD) scheme for the wireless sensor networks. This scheme takes the advantage of the deployment knowledge and the group membership of its neighbors, available in many sensor network applications. This information is then utilized to find out whether the estimated location is consistent with its observations. In case of an inconsistency LAD would report an anomaly.

Loo *et al.* [3] have proposed an anomaly based intrusion detection scheme that is used to detect network level intrusions, e.g., routing attacks. They use clustering algorithm to build the model of normal network behavior, and then use this model to detect anomalies in traffic patterns. IDS will be installed on each sensor and each IDS will function independently.

Da Silva *et al.* [5] have proposed high level methodology to construct the decentralized IDS for wireless sensor networks. They have adopted statistical approach based on the inference of the network behavior. The network behavior is obtained from the analysis of the events detected at the specific monitor node, which is responsible for monitoring its one-hop neighbors looking for intruder(s).

Liu *et al.* [6] have proposed insider attack detection scheme for wireless sensor networks. They have adopted localized distributed and cooperative approach. This scheme explores the spatial correlation in neighborhood activities and requires no prior knowledge about normal or malicious nodes. This scheme works in four phases: (1) collection of local information about neighborhood nodes (e.g., packet dropping rate, sending rate, etc.), (2) filtering the collected data, (3) identification of initial outlying (malicious) nodes, and (4) applying majority vote to obtain a final list of malicious nodes. Once the node detects some malicious node, it will forward the report to the base station. Afterwards the base station will isolate that node from the network.

### 3.2. Intrusion Prevention Schemes

Su *et al.* [8] have proposed an energy-efficient Hybrid Intrusion Prohibition (eHIP) system for cluster-based wireless sensor networks. The eHIP system consists of two subsystems: Authentication-based Intrusion Prevention (AIP) subsystem and Collaboration-based Intrusion Detection (CID) subsystem. In AIP, two distinguish authentication mechanisms are proposed to verify the control and sensed data messages with the help of HMAC and the modified form of one-key chain [13] mechanisms. CID is also consisted of two subsystems: cluster head monitoring (CHM) system and member node monitoring (MNM) system. In CHM, all member nodes are divided into multiple monitoring groups. With respect to security requirements, each monitoring group has various number of monitoring nodes. Every monitoring group monitors the cluster head. Whenever any monitoring group detects malicious activity of the cluster head, it generates an alarm that is forwarded to all member nodes of the cluster. Each member node maintains the alarm table. If the number of alarms exceeds then the alarm threshold, the cluster head will be declared as a malicious node. The member node monitoring mechanism is performed at the cluster head and limited to the detection of compromised nodes through the used pair-wise key only.

Zhang *et al.* [9] have proposed a nice application-independent framework for identifying compromised nodes. This framework is based on alerts generated by specific intrusion detection system. The authors have adopted a centralized approach and used a simple graph theory. However, this scheme has some limitations, e.g., it provides some late detection of compromised nodes, because the detection process will always start at the end of each time window. If the size of the time window is large (e.g., one hour, as mentioned in [9]), then it is very likely that an adversary can achieve its objective during that time window. If the time window is small, then the result may not be accurate. Also, the detection accuracy is mainly dependent on the size of the network density. If the network size decreases, then the detection accuracy will also decrease.

## 4. Network Model, Assumptions and Definitions

### 4.1. Network Model and Assumptions

Sensor nodes are deployed in an environment either in a random fashion or in a grid fashion. After deployment nodes become static, nodes are organized into clusters. The reason behind taking cluster-based network model is that it is widely used in real world scenarios for efficient network organization [14]. Within a cluster, communication mechanism could be single-hop [15] or multi-hop [16]. In case of a multi-hop clustering environment, the cluster could be divided into two or three sensor sub-clusters for the purpose of distributed detection [17].

We assume that any cooperative-based distributed anomaly detection or IDS is already deployed in the WSNs and forwards claims to the other node(s) whenever it detects anomalies or intrusions. Based on the mechanism of the IDS, every node or subset of nodes (within a cluster) acts as a monitoring node. The malicious node must fall into the radio range of the monitoring node. And the node (who received the claim from the monitoring node) has the knowledge about the neighboring nodes of the monitoring and malicious nodes (the malicious and monitoring nodes belong to the same cluster). Within a cluster or sub-cluster, all monitoring nodes can communicate with each other directly. We also assume that the multiple sensor nodes in a neighborhood can sense the same anomaly/intrusion. We also assume that all information is exchanged in a secure encrypted manner. For this purpose, every monitoring node share a unique secret key [18] with other monitoring node(s) that are in the same cluster.

### 4.2. Definitions

A legitimate node compromised by an adversary is called a malicious node. In order to hide the presence of the adversary, a malicious node could also perform all activities of the normal nodes, such as monitoring, ciphering of data, forwarding of packets, etc.

**Figure 2.**
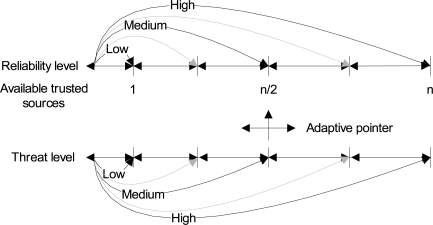
Intrusion-aware reliability mode concept.

Reliability means the confidence level on a certain decision. It can simply be categorized into three levels: (1) low, (2) medium, and (3) high. At low reliability, validation is based on the confirmation from any single available trusted source. At medium reliability, validation is based on the confirmation from half of the available trusted sources. At high reliability, validation is based on the confirmation from all of the trusted sources. In general, the reliability level (*R_L_*) is define as:

(1) $$\begin{array}{ll} R_{L}=q\;\;\;; & q\leq n \end{array}$$

where *n* represents the total number of available trusted nodes, and *q* represents the number of nodes consulted. However, in order to achieve more flexibility and adaptability, we have adopted the intrusion-aware reliability mode concept, in which the validation is based on the level of a threat of an anomaly or intrusion. This approach will also reduce the communication cost as described in Section 6.1. Threats could also be categorized into low, medium, high or other. Depending on the level of the threat, intrusion-aware reliability mode is set to low, medium, high or other, as shown in Figure 2.

## 5. Intrusion-aware Alert Validation Algorithm

Our proposed intrusion-aware alert validation algorithm execute on sender as well as on receiver monitoring nodes. Sender monitoring nodes are mainly responsible for the detection of malicious nodes and generation of alert messages, whereas receiver monitoring nodes are mainly responsible for the validation of alert messages. Both sender and receiver nodes go through different phases as described below.

### 5.1. Sender Monitoring Node

**Figure 3.**
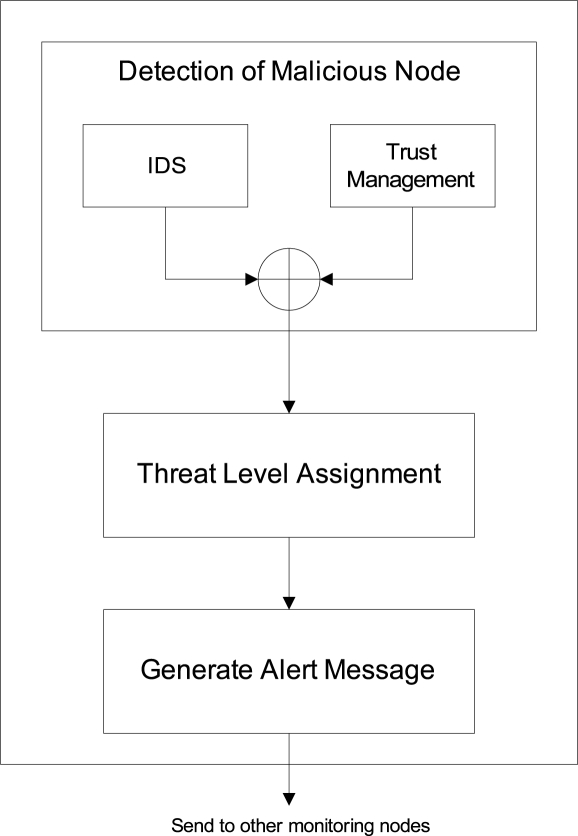
Intrusion-aware validation algorithm at sender end.

In our proposed algorithm, sender monitoring node will perform mainly three steps (as shown in Figure 3): (1) detection of malicious node, (2) threat level assignment, and (3) generation of alert message.

#### Phase 1: Detection of Malicious Node

A node can be classified into one of the three categories [19]: trustworthy, untrustworthy, and uncertain. A node is considered to be trustworthy if it interacts successfully most of the time with the other nodes. A node is considered untrustworthy if it tries to do as many unsuccessful interactions as possible with the other nodes. An untrustworthy node could be a faulty [20] or malicious node. A node is considered uncertain if it performs both successful and unsuccessful interactions. Detailed definition of the successful and unsuccessful interactions and trust calculation methodology is available in our paper [21] and provided here in a simplified form.

A sender will consider an interaction successful if the sender receives confirmation that the packet is successfully received by the neighbor node and forwarded towards the destination in an unaltered fashion. The first requirement of successful reception is achieved on the reception of the link layer acknowledgment (ACK). The second requirement of forwarding towards the destination is achieved with the help of enhanced passive acknowledgment (PACK) by overhearing the transmission of a next hop on the route, since they are within the radio range [22]. If the sender node does not overhear the retransmission of the packet within a threshold time from its neighboring node or the overheard packet is found to be illegally fabricated (by comparing the payload that is attached to the packet), then the sender node will consider that interaction as unsuccessful.

With the help of this simple approach, several attacks can be prevented, i.e., the black hole attack is straightforwardly detected when malicious node drops the incoming packets and keeps sending self-generated packets [23]. Similarly, sink hole attack [24], an advanced version of the black hole attack, is also easily detectable by looking at the passive acknowledgment. Likewise, the selective forwarding attack [25] and gray-hole attack [26] can also be eliminated with the aid of above mentioned approach.

Based on these successful and unsuccessful interactions, node *x* can calculate the trust value of node *y*:

(2) $$T_{x,y}=\left[100\;\left(\frac{S_{x,y}}{S_{x,y}+U_{x,y}}\right)\;\left(1-\frac{1}{S_{x,y}+1}\right)\right]$$

where [.] is the nearest integer function, *S_x,y_* is the total number of successful interactions of node *x* with *y* during time Δ*t*, *U_x,y_* is the total number of unsuccessful interactions of node *x* with *y* during time Δ*t*. After calculating trust value, a node will quantize trust into three states as follows:

(3) $$\mathrm{Mp}\left(T_{x,y}\right)=\left\{\begin{array}{cc} \text{trustworthy} & 100-f\leq T_{x,y}\leq 100\\ \text{uncertain} & 50-g\leq T_{x,y}<100-f\\ \text{untrustworthy} & 0\leq T_{x,y}<50-g \end{array}\right\}$$

where, *f* represents half of the average values of all trustworthy nodes and *g* represents one-third of the average values of all untrustworthy nodes. Both *f* and *g* are calculated as follows:

(4) $$f_{j+1}=\{\begin{array}{cc} \left[\frac{1}{2}\left(\frac{\sum_{i\in R_{x}}T_{x,i}}{\left|R_{x}\right|}\right)\right] & 0<\left|R_{x}\right|\leq n-1\\ f_{j} & \left|R_{x}\right|=0 \end{array}$$

(5) $$g_{j+1}=\{\begin{array}{cc} \left[\frac{1}{3}\left(\frac{\sum_{i\in M_{x}}T_{x,i}}{\left|M_{x}\right|}\right)\right] & 0<\left|M_{x}\right|\leq n-1\\ g_{j} & \left|M_{x}\right|=0 \end{array}$$

where [.] is the nearest integer function, *R_x_* represents the set of trustworthy nodes for node *x*, *M_x_* the set of untrustworthy nodes for node *x*, and *n* is the total number of nodes that contains trustworthy, untrustworthy and uncertain nodes. The initial trust values of all nodes are 50, which represents the uncertain state. Initially *f* and *g* are equal to 25 and 17 respectively, although other values could also be used by keeping the following constraint intact: *f_i_* − *g_i_* ≥ 1, which is necessary for keeping the uncertain zone between a trusted and untrustworthy zone. The values of *f* and *g* are adaptive. During the steady-state operation, these values can change with every passing unit of time which creates dynamic trust boundaries. At any stage, when |*R_x_*| or |*M_x_*| becomes zero, the value of *f_j_*_+1_ or *g_j_*_+1_ remains the same as the previous values (*f_j_* and *g_j_*). The nodes whose values are above 100 − *f* will be declared as trustworthy nodes (Equation 3), and nodes whose values are lower than 50 − *g* will be consider as untrustworthy nodes (Equation 3). After each passage of time, Δ*t*, nodes will recalculate the values of *f* and *g*. This trust calculation procedure will continue in this fashion.

The time window length (Δ*t*) could be made shorter or longer based on the network analysis scenarios. If Δ*t* is too short, then the calculated trust value may not reflect the reliable behavior. On the other hand, if it is too long, then it will consume too much memory to store the interaction record at the sensor node. Therefore, various paremters can be used to adjust the length of Δ*t*. In simplicity, let us assume a cluster size, *n*, as the single paramters; then, Δ*t* is equal to *n* − 1. This approach reduces the problems associated with too short or too long time window lengths. Moreover, the time window lengths are adaptive based on the cluster size. If the size of the cluster changes, then the time window length will also change.

#### Phase 2: Threat Level Assignment

Whenever a monitoring node detects malicious node, it generates an alert. This alert will be forwarded to the other neighbor monitoring nodes. In our proposed algorithm, an alert message also contains the information about intensity or level of threat. This level of threat is calculated based on the trust value of the malicious node. The lesser the trust value, the higher the threat level. For example, if a node is continuously dropping all incoming packets (black hole attack), then based on the trust management methodology defined above, the trust value of a malicious node becomes zero. So, the level of threat for this kind of attack is high. If a node is performing sink hole attack, then the trust value of a node become higher than the node performing the black hole attack. Therefore, the threat level is less as compared with the earlier one.

Based on the trust value of a malicious node, a node will quantize threat level (*H_level_*) in following way:

(6) $$H_{\mathrm{level}}=\left\{\begin{array}{cc} H_{1} & (k-1)\times \frac{50-g}{k}\leq T_{\mathrm{mal}}\leq 50-g\\ H_{2} & (k-2)\times \frac{50-g}{k}\leq T_{\mathrm{mal}}<(k-1)\times \frac{50-g}{k}\\ \vdots & \vdots \\ H_{k-1} & \frac{50-g}{k}\leq T_{\mathrm{mal}}<2\times \frac{50-g}{k}\\ H_{k} & 0\leq T_{\mathrm{mal}}<\frac{50-g}{k} \end{array}\right\}$$

where *k* represents total number of threat levels, 50 − *g* represent the upper limit of the untrustworthy zone as defined in Equation 3. *T_mal_* represent the trust value of a malicious node. Let us assume that there are three threat levels (*k*=3): low, medium and high. In that case, a node will quantize threat level (*H_level_*) in following way:

(7) $$H_{\mathrm{level}}=\left\{\begin{array}{cc} \text{Low} & 2\times \frac{50-g}{3}\leq T_{\mathrm{mal}}\leq 50-g\\ \text{Medium} & \frac{50-g}{3}\leq T_{\mathrm{mal}}<2\times \frac{50-g}{3}\\ \text{High} & 0\leq T_{\mathrm{mal}}<\frac{50-g}{3} \end{array}\right\}$$

The concept of threat level is later used in our algorithm for a selection of an appropriate reliability level.

#### Phase 3: Generation of Alert Message

Once the threat level is assigned, a node will generate an alert/claim message. This message contains four types of information.

1. Identity of the sender node (*ID_sender_*).
2. Identity of the malicious node (*ID_mal_*).
3. Threat level (*H_level_*).
4. Threat detail, like code etc.

This message will be forwarded to the other monitoring nodes.

### 5.2. Receiver Monitoring Node

**Figure 4.**
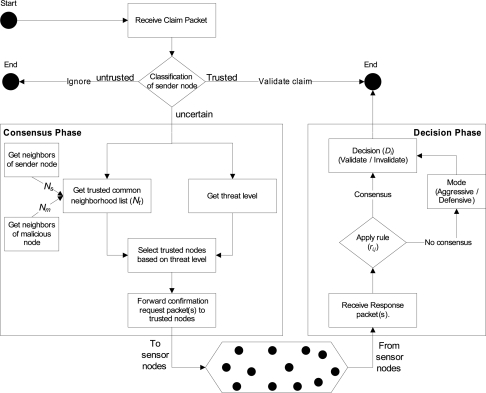
Intrusion-aware validation algorithm at the receiver end.

Intrusion-aware alert validation algorithm at the receiver end is shown in Figure 4. It shows that, if the claim packet is received from *trustworthy* monitoring node, then claim will be validated straightforwardly. If the claim packet is received from the *untrustworthy* monitoring node, then no consideration will be given to that claim packet. If the claim packet is received from the *uncertain* monitoring node, then our proposed intrusion-aware validation algorithm goes through two phases: (1) consensus phase, and (2) decision phase. Details about these phases are given below.

#### Phase 1 (Consensus Phase)

Whenever a designated node receives the claim/alert packet, it first checks (1) if the sender is uncertain, and (2) if the identity of a new malicious node is already declared as a malicious node (Algorithm 1, Line 1:2). If not, then the node will first get the common neighborhood list (*N_sm_*) of the sender and malicious nodes respectively (Line 3:5). Afterwards, the node will perform filtering by eliminating any known malicious node(s) from that list (Line 6). Based on the threat level, confirmation request packet(s) is forwarded to randomly selected node(s) from the *N_t_* list (Line 7:19). For example, if the threat level is low, then the confirmation request is forwarded to the one randomly selected trustworthy node from the list *N_t_* (Line 8:9). If the threat level is medium, then the confirmation request packet is forwarded to half of the randomly selected trustworthy nodes from the list *N_t_* (Line 10:13). If the threat level is high, then the confirmation request packet is forwarded to all trustworthy nodes in the list *N_t_* (Line 14:17). If the information about the malicious node is already present (Line 20), then the node will just update its old record (Line 21).

**Algorithm 1:** Phase 1: Consensus Phase

1: Received Claim Packet (*ID_sender_, ID_mal_, H_level_*, detail);
2: **if** *ID_sender_* is uncertain and *ID_mal_* is new **then**
3: *N_s_* = GetNeighorList(*ID_sender_*);
4: *N_m_* = GetNeighorList(*ID_mal_*);
5: *N_sm_* = *N_s_* ∩ *N_m_*;
6: *N_t_* = Eliminate_Known_Malicious_Nodes(*N_sm_*);
7: **if** *N_t_ ≠* = *ϕ***then**
8: **if** ThreatLevel(*T H_level_*) is Low **then**
9: Send conf_req_pkt(*rand*(*N_t_*)*, ID_mal_, H_level_*,det);
10: **else if** ThreatLevel(*T H_level_*) is Medium **then**
11: **for** *i* = 1 *to len*(*N_t_*)/2 **do**
12: Send conf_req_pkt(*rand*(*N_t_*)*, ID_mal_, H_level_*,det);
13: **end for**
14: **else**
15: **for** *i* = 1 *to len*(*N_t_*) **do**
16: Send conf_req_pkt(*ID_i_, ID_mal_*,det);
17: **end for**
18: **end if**
19: **end if**
20: **else**
21: Update Record;
22: **end if**

#### Phase 2 (Decision Phase)

Once the confirmation request packet(s) is forwarded to the particular node(s) then the phase 2 of the validation algorithm is triggered. In this phase algorithm will first wait for the confirmation response packets until *δt* time, where *δt* is calculated as:

(8) $$\delta t=2\;\left[2t_{\mathrm{prop}}+t_{\mathrm{proc}}\right]$$

Here, *t_prop_* is the propagation time between the requester and farthest responder (in terms of hops or geographical location) among nodes where the request packets were forwarded. The *t_proc_* is the estimated processing time of the request at the responder end.

A node will expect three types of responses (*r*) from the nodes where confirmation request packets were forwarded:

(9) ri,j={1ifagree with claim0ifdon't know−1ifnot agree with claim

where *r_i,j_* represents that the node *i* received the response packet from the node *j* and *j ∈ N_t_*. A node *i* will make the decision (*D*) about the validity and invalidity of the claim based on the following rule:

(10) $$D_{i}=\{\begin{array}{ccc} \text{validate} & \text{iff} & \sum_{j=0}^{n_{\mathrm{res}}}r_{i,j}>0\\ \text{no consensus} & \text{iff} & \sum_{j=0}^{n_{\mathrm{res}}}r_{i,j}=0\\ \text{invalidate} & \text{iff} & \sum_{j=0}^{n_{\mathrm{res}}}r_{i,j}<0 \end{array}$$

where *n_res_* represents the total number of the response packets received by the node *i* in response to the number of the request packets (*n_req_*). Here 0 ≥ *n_res_ ≤ n_req_*.

If the claim is invalidated, then the sender of the claim will declare it as a malicious node. That helps to provide protection against any possible security threats, such as flooding, denial of service attacks, etc.

If no consensus is available, then the algorithm will decide based on its mode that is set by the administrator. There are two types of modes: aggressive and defensive. If the algorithm is set in the aggressive mode, then the node will validate the claim; if it is set in the defensive mode, then the node will invalidate the claim.

**Responder monitoring nodes:** Whenever any monitoring node receives confirmation request packet for alert validation, it will first check the status of the sender. If the sender is trusted, it will generate confirmation response packet and will not generate the same alert if responder node agree with the claim. Also, the responder node will update its malicious node list. If the responder node receives the same alert message from another monitoring node, it will straightforwardly validate that claim. This approach helps to suppress any extra requests for the same alert.

**Tolerance for false alarm:** In our proposed algorithm, default tolerance level for false alarms generated by any node is zero. As mentioned earlier, if a claim is invalidated, the sender of the claim will be declared as a malicious node. If we do not declare the sender as a malicious node, then it may result in flooding or denial of service attacks. However, if we declare the sender as a malicious node, it may cause a node to be evicted from the network due to false alarm.

In order to solve the above problem, we can introduce a tolerance level metric in our algorithm. Tolerance level determines the amount of traffic each node can generate for the claims about which it is unsure. Tolerance level will depend on network capacity and node abundance. It may also depend on the energy level of the network. If the energy level is too low, the application can decide not to tolerate any such traffic.

## 6. Analyses and Evaluation

### 6.1. Communication Overhead Analysis

The communication overhead of the validation algorithm is dependent on three factors: (1) total number of intrusion claims (*I_c_*), (2) number of commonly trusted neighboring nodes, and (3) threat level of intrusion or anomaly. Table 2 shows the communication overhead, in which *m_t_* represents the average number of trusted common neighboring nodes between the monitoring and malicious nodes and *I_l_, I_m_*, and *I_h_* represents the total number of low, medium and high intrusion level threats respectively. Here *I_c_* = *I_l_* + *I_m_* + *I_h_*.

**Figure 5.**
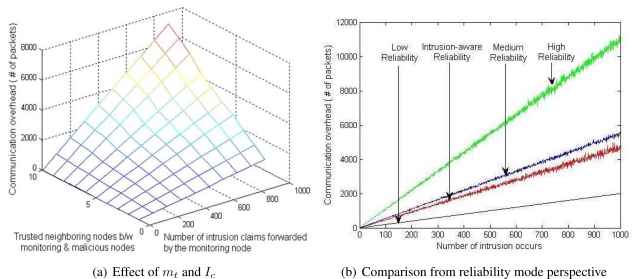
Average communication overhead of validation algorithm after 1000 simulation runs in which different levels of intrusions occurs randomly.

**Figure 6.**
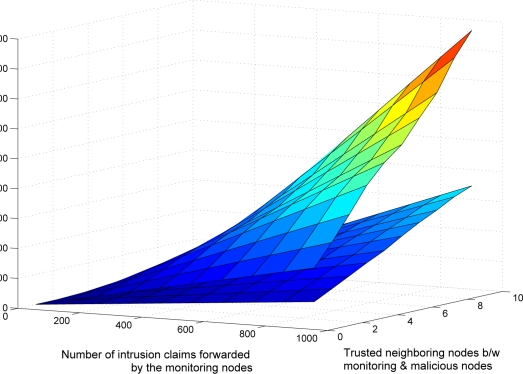
Effect of false alarm tolerance factor on communication.

Figure 5 shows the average communication overhead (1,000 simulation runs) of the proposed validation algorithm. During the simulation, different levels (low, medium or high) of threats of anomalies and intrusions occur randomly. Figure 6(a) shows the effect of average number of commonly trusted neighboring nodes (between the monitoring and the malicious nodes) *m_t_* and the total number of intrusions *I_c_* occurred in the network. It shows that as the number of *m_t_* or *I_c_* increases, the communication overhead of the validation scheme also increases linearly. Figure 6(b) shows the comparison between the four different levels of the reliability modes. In the simulation, each monitoring node has a random number of commonly trusted neighboring nodes. This figure shows that the intrusion-aware reliability mode introduces less communication overhead then the medium and high level reliability modes. At a modest communication cost, it provides adequate reliability required by the nature of the intrusion claim.

Figure 6 shows the effect of tolerance level for false alarms on communication overhead. As we mentioned earlier, the default tolerance level in our proposed scheme is zero. During simulation, we introduce four tolerance levels (0–3) that occurred randomly. Figure 6 shows that as the number of *m_t_* or *I_c_* increases, the communication overhead of the validation scheme (with random tolerance) increases more sharply as compared with the zero tolerance level.

### 6.2. Reliability Analysis

If we assume that the responding node have equal probability of sending any one of the three possible responses (agree, disagree and don’t know), then the total probability (*P_c_*) of a algorithm to reach at the consensus state (validate or invalidate) is:

(11) $$P_{c}=\frac{N_{c}}{K^{n_{\mathrm{res}}}}$$

where *N_c_* represents the number of nodes reaching a consensus and *K* represents the number of possible outcomes (agree, disagree and don’t know) produces by the node. If the probability distribution is not uniform between possible outcomes, then the total probability (*P_c_*) of an algorithm to reach the consensus state (validates or invalidate) is:

(12) $$\begin{array}{l} P_{c}=\sum_{m=1}^{M}\left(\prod_{i=1}^{n_{\mathrm{res}}}{\mathrm{PMF}}_{i}\left(S_{m}(i)\right)\right)\times \delta (m)\\ \text{where}\;M=K^{n_{\mathrm{res}}} \end{array}$$

where *δ*(*m*) is one if *m* node reaches the consensus, zero if otherwise. *PMF_i_* is the probability mass function that captures the probability distribution of the symbol produced by the node *i. S_m_*(*i*) is the *i^th^* symbol in the *m^th^* node result. More details and derivation of these two probability equations are given in [27].

**Figure 7.**
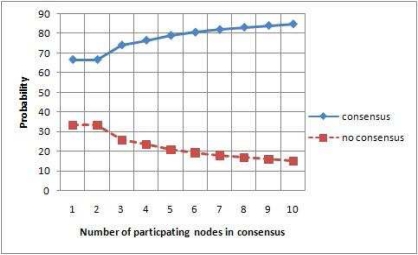
Probability of reaching at consensus and no consensus state.

Figure 7 shows the simulation result for the probability of reaching consensus (validate or invalidate) of our validation algorithm. It shows that as the number of participating nodes increases in the consensus process, the probability of reaching some consensus also increases linearly.

### 6.3. Security Resiliency Analysis

Let’s denote the claimant node (the sender), let **m** denote the node accused of being malicious and let **a** denote the node that receives this claim from **s** about **m**. As before, let *N_t_* denote the filtered list of nodes obtained after performing Line 6 of the algorithm for the consensus phase. From a security point of view, we consider 4 possible events:

1. Event S: **s** sends a true claim.
2. Event S̄: The complement of event S.
3. Event N: All nodes in *N_t_* send correct responses.
4. Event N̄: A non-empty subset of nodes in *N_t_* send incorrect responses.

Notice that there is no question of **a** behaving maliciously since the claim received by it is for its own benefit. Notice further that by incorrect response, we mean nodes responding with −1, where the right answer should be either 1 or 0. Denote by M the event that **a** decides that **m** is malicious. We are interested in the four resulting conditional probabilities. We calculate them sequentially in the following. For the ease of analysis, we assume that if **a** comes to no consensus, it will take the claim of **s** as true.

**Claim 1:** Let *m* be the number of nodes in *N_t_* that agree with the claim of **s**. Then $\text{Pr}\;\left[M|S,N\right]=\left(\begin{array}{c} N_{t}\\ m \end{array}\right)\sum_{i=1}^{m}\frac{\left(\underset{i}{N_{t-m}}\right)}{2^{N_{t-m}}}$ when *m < N_t_*/2 and 1 otherwise.

*Proof:* First assume that *m < N_t_*/2. The remaining nodes in *N_t_* will either send −1 or 0 as the response. Event M will be true if the sum of the −1’s is less than or equal to *m*. Assuming *m* to be fixed, this probability is:

$$\sum_{i=1}^{m}\frac{\left(\underset{i}{N_{t-m}}\right)}{2^{N_{t-m}}}$$

Out of *N_t_* nodes, $\left(\begin{array}{c} N_{t}\\ m \end{array}\right)$ is the total number of ways in which *m* nodes can agree with the claim. So the probability is then:

$$\left(\begin{array}{c} N_{t}\\ m \end{array}\right)\sum_{i=1}^{m}\frac{\left(\underset{i}{N_{t-m}}\right)}{2^{N_{t-m}}}$$

The case when *m* ≥ *N_t_*/2 is obvious.

**Claim 2:** Let *m*^′^ be the number of nodes in *N_t_* that send false responses. Let *m* be the number of nodes in *N_t_* that agree with the claim of **s**. Then $\text{Pr}\;\left[M|S,\bar{N}\right]=\left(\begin{array}{c} N_{t}\\ m-m^{\prime } \end{array}\right)\sum_{i=1}^{m-m^{\prime }}\frac{\left(\underset{i}{N_{t-m+m^{\prime }}}\right)}{2^{N_{t-m+m^{\prime }}}}$ when *m* ≥ *m*^′^ and 0 otherwise. In particular, Pr [ M| S, N̄] = 0 if *m*^′^ > *N_t_*/2.

*Proof:* This is analogous to Claim 1, with the exception that now *m* has to be greater than at least *m*^′^, since otherwise the sum of responses will be less than 0. Hence we replace *m* by *m* − *m*^′^ in the probability obtained from Claim 1. The special case when *m*^′^ > *N_t_*/2 is obvious since then the sum of the responses will always be less than 0.

**Claim 3:** Pr[ M| S̄, N] = 1 − Pr [ M| S, N].

*Proof:* Since now the number of nodes that agree with **s** will play an opposite role, the result follows.

**Claim 4:** Let *m*^′^ be the number of nodes in *N_t_* that send false responses. Let *m* be the number of nodes in *N_t_* that agree with the claim of **s**. Then $\text{Pr}\;\left[M|\bar{S},\bar{N}\right]=\left(\begin{array}{c} N_{t}\\ m+m^{\prime } \end{array}\right)\sum_{i=1}^{m+m^{\prime }}\frac{\left(\underset{i}{N_{t-m-m^{\prime }}}\right)}{2^{N_{t-m-m^{\prime }}}}$ when *m* + *m*^′^ < *N_t_*/2 and 1 otherwise.

*Proof:* This is analogous to the proof of Claim 1. Notice that now there are a total number of *m* + *m*^′^ nodes that agree with **s**. Thus we simply replace *m* by *m* + *m*^′^ to complete the proof.

Finally we look at the event when **a** marks **s** as malicious. This will happen if **a** comes to a consensus opposite to the claim of **s**. Let this event be denoted as O. We are interested in Pr [ O| S] and Pr [ O| S̄]. Let *p* = Pr [ N]. We have the following straightforward result:

**Claim 5:** We have:

$$\begin{array}{l} \text{Pr}\;[O|S]=p(1-\text{Pr}\;[M|S,\;N])+(1-p)(1-\text{Pr}\;[M|S,\;\bar{N}])\\ \text{Pr}\;[O|\bar{S}]=p(1-\text{Pr}\;[M|\bar{S},\;N])+(1-p)(1-\text{Pr}\;[M|\bar{S},\;\bar{N}]) \end{array}$$

The results that we obtain above are an upper bound on the adversary’s limitations. This analysis provides a general probability method for the determination of certain security metric.

## 7. Conclusion and Future Work

Existing cooperative-based distributed anomaly and intrusion detection schemes of WSNs do not provide assurance that the reports/alerts/claims received by the other node(s) were really sent by the trusted legitimate node(s). Therefore, in this paper we have proposed the first validation algorithm for trusting anomalies and intrusion claims. This algorithm uses the concept of an intrusion-aware reliability parameter that helps to provide adequate reliability at a modest communication cost.

The proposed work is based on a few strict assumptions, i.e., multiple nodes can sense same anomaly or intrusion. In practice, it is quite possible that only one node can detect some specific anomaly or intrusion. Our proposed scheme does not adequetly deal with this case. Therefore, more work is needed to make the proposed scheme further flexible.

## Figures and Tables

**Table 1.** Summarization of proposed Anomalies and IDS schemes of WSNs

		[1]	[2]	[3]	[4]	[5]	[6]
Classification	Technique	Signature-based	Statistical-based	Statistical-based	Statistical-based	Statistical-based	Statistical-based
	Architecture	Distributed & cooperative	Distributed & cooperative	Distributed & uncooperative	Hybrid	Distributed & uncooperative	Distributed & cooperative
Specifications	Installation of IDS	Each sensor node	Each sensor node	Each sensor node	Each primary node of a group	Special monitor nodes in network	Each sensor node
	IDS Scope	Multilayer (Appl., Net., MAC & Phy.)	Application layer	Network layer	Application layer	Multilayer (Appl., Net., MAC & Phy.)	Network layer
	Attacks detects	Masquerade attack, and forged packets attacks	Localization anomalies	Routing attacks e.g., Periodic error route attack, active & passive sinkhole attack	Correlated anomalies / attacks (invalid data insertion)	Worm hole, data alteration, selective forwarding, black hole, & jamming	Routing attacks e.g., packet dropping etc.
Network	Sensor node	Static / Mobile	Static	Static / Mobile	Static / Mobile	Static	Static
	Topology	Any	Any	Any	Cluster-based	Tree-based	Any

**Table 2.** Communication overhead of reliability modes.

	Cost
Low	2*I_c_*
Medium	*m_t_I_c_*
High	2*m_t_I_c_*
Intrusion-aware	2*I_l_* + (*I_m_* + 2*I_h_*)*m_t_*
